# Extracellular Vesicles Derived from Human Gingival Mesenchymal Stem Cells: A Transcriptomic Analysis

**DOI:** 10.3390/genes11020118

**Published:** 2020-01-21

**Authors:** Serena Silvestro, Luigi Chiricosta, Agnese Gugliandolo, Jacopo Pizzicannella, Francesca Diomede, Placido Bramanti, Oriana Trubiani, Emanuela Mazzon

**Affiliations:** 1 IRCCS Centro Neurolesi “Bonino-Pulejo”, 98124 Messina, Italy; serena.silvestro@irccsme.it (S.S.); luigi.chiricosta@irccsme.it (L.C.); agnese.gugliandolo@irccsme.it (A.G.); placido.bramanti@irccsme.it (P.B.); 2 ASL02 Lanciano-Vasto-Chieti, “Ss. Annunziata” Hospital, 66100 Chieti, Italy; jacopo.pizzicannella@unich.it; 3 Department of Medical, Oral and Biotechnological Sciences, University “G. d’Annunzio” Chieti-Pescara, 66100 Chieti, Italy; francesca.diomede@unich.it (F.D.); trubiani@unich.it (O.T.)

**Keywords:** extracellular vesicles, human gingival mesenchymal stem cells, next generation sequencing, transciptome

## Abstract

Human gingival mesenchymal stem cells (hGMSCs) have outstanding characteristics of proliferation and are able to differentiate into osteogenic, chondrogenic, adipogenic, and neurogenic cell lineages. The extracellular vesicles (EVs) secreted by hGMSCs contain proteins, lipids, mRNA and microRNA have emerged as important mediators of cell-to-cell communication. In this study, we analyzed the transcriptome of hGMSCs-derived EVs using Next Generation Sequencing (NGS). The functional evaluation of the transcriptome highlighted 26 structural protein classes and the presence of “non-coding RNAs”. Our results showed that EVs contain several growth factors such as Transforming Growth Factor-β (TGF-β), Fibroblast Growth Factor (FGF), and Vascular Endothelial Growth Factors (VEGF) implicated in osteoblast differentiation and in angiogenetic process. Furthermore, the transcriptomic analysis showed the presence of glial cell-derived neurotrophic factor (GDNF) family ligands and neurotrophins involved in neuronal development. The NGS analysis also identified the presence of several interleukins among which some with anti-inflammatory action. Moreover, the transcriptome profile of EVs contained members of the Wnt family, involved in several biological processes, such as cellular proliferation and tissue regeneration. In conclusion, the huge amount of growth factors included in the hGMSCs-derived EVs could make them a big resource in regenerative medicine.

## 1. Introduction

The oral cavity has been identified as an easily-accessible reservoir of human mesenchymal stem cells (hMSCs) [1,2]. The oral hMSCs were successfully isolated and characterized from a variety of oral tissues, including dental pulp [3], apical papilla [4], exfoliated deciduous teeth [5], dental follicle [6], periodontal ligament [7], and gingiva [8]. Oral stem cells originate from neural crests and represent a transient population of embryonic pluripotent stem cells [9,10]. Among oral derived hMSCs, human gingival MSCs (hGMSCs) show a self-renewal ability and a fast proliferation rate. Several studies report a multilineage differentiation ability of hGMSCs into osteoblastic, adipocytic, chondrocyte, endothelial, and neural directions [11]. Moreover, the hGMSCs show spindle-like cell morphology and plastic adherence [12,13,14]. For standardization purposes, studies commonly refer to the marker arrangement proposed by the International Society for Cellular Therapy (ISCT) for hGMSCs’ identification [15]. In line with the evidence present in literature, our research group, using the cytofluorimetric evaluation, has already demonstrated that our hGMSCs show an expression profile characterized by specific cell surface markers, such as CD29, CD44, CD73, CD90, and CD105, and stemness associated markers, as OCT3/4, SSEA4, and SOX2 [16,17].

The striking positive attributes of hGMSCs make them attractive cellular sources in the field of tissue engineering regenerative medicine for several therapeutic applications such as bone defects regeneration, skin wound repair, periodontal regeneration, rheumatoid arthritis, and other autoimmune diseases [14].

The hGMSCs exert their therapeutic effects also through the release of extracellular vesicles (EVs) [18]. The EVs are small membrane vesicles containing abundant proteins, lipids, a pool of soluble cytokines and nucleic acids such as mRNA and microRNA [19]. EVs represent intercellular communication systems able to interact with target cells by binding to the cell surface receptors, transferring membrane proteins, merging their membrane contents into cell recipient cell plasma membrane [20,21]. In this regard, the EVs isolated from different cell sources represent a new tool for a regenerative and therapeutic approach in tissue regeneration applications [22].

The positive involvement of the EVs suggests the inclusion of several transcripts involved in various basic biological processes. Therefore, in this study, we want to analyze the content of hGMSC-derived EVs. For this reason, we inspected the transcriptomic profile of the hGMSC-derived EVs using NGS analysis in order to evaluate the family of the transcripts that are included and therefore understand their potential therapeutic efficacy in the field of regenerative strategy.

## 2. Materials and Methods

### 2.1. hGMSCs Culture Estabilishment

All subjects gave their informed consent for inclusion before they participated in the study. The study was conducted in accordance with the Declaration of Helsinki, and the protocol was approved by the Medical Ethics Committee at the Medical School, “G. d’Annunzio” University, Italy (n°266 17 April 2014, Principal Investigator: Trubiani Oriana). Gingival tissue biopsies were obtained from six healthy adult volunteers with no gingival inflammation during teeth removal for orthodontic purpose. The gingival specimens were de-epithelialized with a scalpel for the exclusion of most of the keratinocytes resident in the gingival tissue as previously described [23]. After collection, hGMSCs was washed several times with PBS (Lonza) and subsequently cultured using MSCGM-CD medium (Mesenchymal Stem Cell Growth Medium Chemically Defined) (Lonza, Basel, Switzerland) at 37 °C with 5% of CO_2_.

### 2.2. hGMSCs–Derived EVs Isolation

The conditioned medium (CM; 10 mL) after 48 h of incubation were collected from hGMSCs at passage 2. The CM was centrifuged at 3000× *g* for 15 min to eliminate suspension cells and debris. For the EVs extraction we used an ExoQuick TC commercial agglutinant (System Biosciences, Euroclone SpA, Milan, Italy). Briefly, 2 mL ExoQuick TC was added to 10 mL of CM recovered from hGMSCs. The mix was incubated overnight at 4 °C without rotation; one centrifugation step was performed at 1500× *g* for 30 min to sediment the EVs and the pellets were resuspended in 500 μL PBS (Ca^2+^ and Mg^2+^).

### 2.3. RNA Extraction and Transcriptomic Analysis

RNA isolation was executed using the Total Exosome RNA and Protein Isolation Kit (catalog # 4478545; Thermo Scientific, Rockford, IL, USA) according to the manufacturer’s protocol. A final volume of 30 μL RNA solution was collected from each. Eppendorf BioSpectrometer fluorescence was used for measuring RNA quality and concentration.

The library preparation was carried out according to the TruSeq RNA Exome protocol (Illumina, San Diego, CA, USA) following manufacturer instructions. 3 EVs batches were analyzed by RNA sequencing. Briefly, RNA extracted by each sample was fragmented at 94 °C for 8 min and the first strand of cDNA was synthesized using the SuperScript II Reverse Transcriptase (Invitrogen, Milan, Italy). After the second strand of cDNA was synthesized and purified by AMPure XP beads (Beckman Coulter, Brea, CA, USA). The 3′ ends of the cDNA were adenylated to allow the adaptor ligation in the following step. After the ligation of indexing adapter, the libraries were purified with AMPure XP beads. A first PCR amplification step was performed to enrich those fragments of DNA that have adaptors on both ends, and also to enhance the quantity of DNA in the library. The library has been validated using the Agilent Technologies 2100 Bioanalyzer. After that, 200 ng of each DNA library were combined and the first hybridization step was performed. In order to eliminate nonspecific binding, magnetic beads coated with streptavidin were used to capture probes hybridized to the target regions, followed by two heated washes to discharge the nonspecific binding from the beads. Then, the enriched libraries were eluted from the beads and were ready for a second cycle of hybridization. This hybridization step was necessary to obtain a wide specificity of regions of capture. After that, the libraries were purified through the AMPure XP bead, and amplified. Libraries were quantified and certified with the Agilent High Sensitivity Kit on a bioanalyzer. Libraries were normalized to 12 pM and subjected to cluster, and single read sequencing was executed on a MiSeq instrument (Illumina) following the protocol guidelines. The produced libraries were loaded for clustering on a MiSeq Flow Cell and then sequenced with a MiSeq Instrument (Illumina). The cluster density validation had been executed by the software of the instrument throughout the run.

The quality of the reads was analysed using the software fastQC (Babraham Institute, Cambridge, UK). The software Trimmomatic (Usadel Lab, Aachen, Germany) was used to remove the adapters and the low-quality bases [24]. Next, the reads were aligned against the reference genome of “Homo Sapiens” available on the University of California Santa Cruz (UCSC) website using Spliced Transcripts Alignment to a Reference (STAR) RNA-seq aligner [25]. Finally, the genes were identified with Cufflinks software (Trapnell Lab, Washington, DC, USA) version 2.2.1 [26]. The genes classification of our dataset was performed using PANTHER [27]. In addition, the last non-cording RNA genes list (release 32) were retrieved by GENCODE website in GTF format. The analysis of the transcripts performed with Cufflinks was matched against this list in order to select all the non-coding genes in our dataset. Finally, the genes from which the transcripts are processed were characterized using the “non-coding RNAs” group (475) from HUGO Gene Nomenclature Committee website. The plots depiction was made with R software.

## 3. Results

### Transcriptomic Investigation of EVs

**Figure 1:**
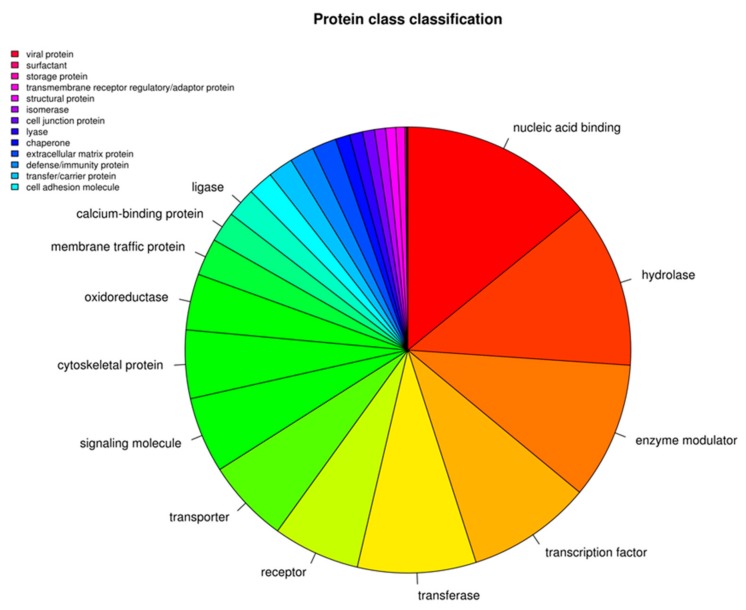
Functional evaluation of the transcriptome performed by Panther. The analysis highlighted the presence of 26 structural proteins classes.

The functional evaluation of the transcriptome performed by Panther highlighted 26 structural protein classes as displayed in the Figure 1. Among the 15,380 genes identified by EVs analysis (Table S1), 15,168 genes are identified by Panther and 8930 hit the “protein class” classification. Specifically, the most represented class is “nucleic acid binding” with 1262 genes able to interact with DNA or RNA. Overrepresented classes are also the hydrolase, the enzyme modulator, the transcription factor, the transferase, the receptor and the transporter with 1067, 886, 808, 768, 562, and 541 genes respectively.

**Figure 2:**
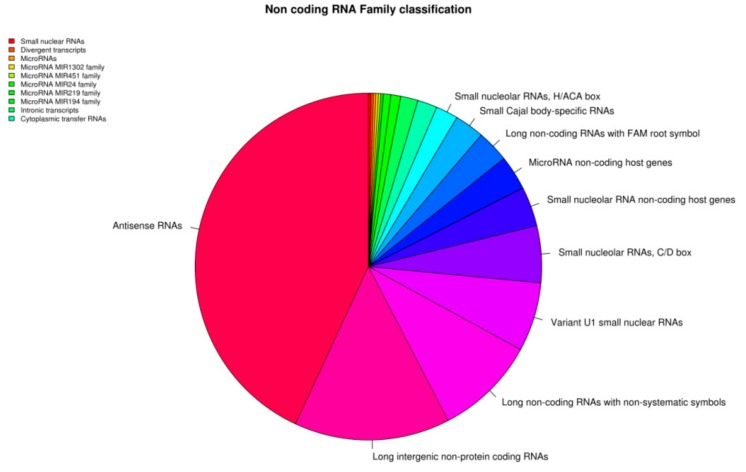
Pie chart representation of the non-coding RNA included in the extracellular vesicles (EVs) transcriptomic profile characterized by HUGO database.

The transcriptomic analysis revealed also 1155 non-coding genes. The “non-coding RNAs” (ncRNAs) classification retrieved by HUGO website characterizes 431 genes in 20 classes (Figure 2). The most represented class is the “Antisense RNAs” in which are included 187 genes involved in the blocking of the mRNA translation process. Overrepresented classes are also the “Long intergenic non-protein coding RNAs” where 63 genes participated as chromatin remodeling or RNA stabilization and 41 genes are “Long non-coding RNAs with non-systematic symbols”; 28 genes belonged to “Variant U1 small nuclear RNAs” that are similar in sequence to U1 small nuclear, involved in the splicing of the pre-mRNA, and 23 genes in “Small nucleolar RNAs, C/D box” that guide chemical modifications and, in particular, methylation. The other classes that included “Small nucleolar RNA non-coding host genes”, “MicroRNA non-coding host genes”, “Long non-coding RNAs with FAM root symbol”, “Small Cajal body-specific RNAsr”, “Small nucleolar RNAs, H/ACA box”, “Small nuclear RNAs”, “Divergent transcripts”, “MicroRNAs”, “MicroRNA MIR1302 family”, “MicroRNA MIR451 family”, “MicroRNA MIR24 family”, “MicroRNA MIR219 family”, “MicroRNA MIR194 family”, “Intronic transcripts”, and “Cytoplasmic transfer RNAs” were less represented. The huge repertoire of ncRNA found, demonstrates that these cells are flooded with these RNAs, which constitute a hidden layer of molecular genetic signals.

**Figure 3:**
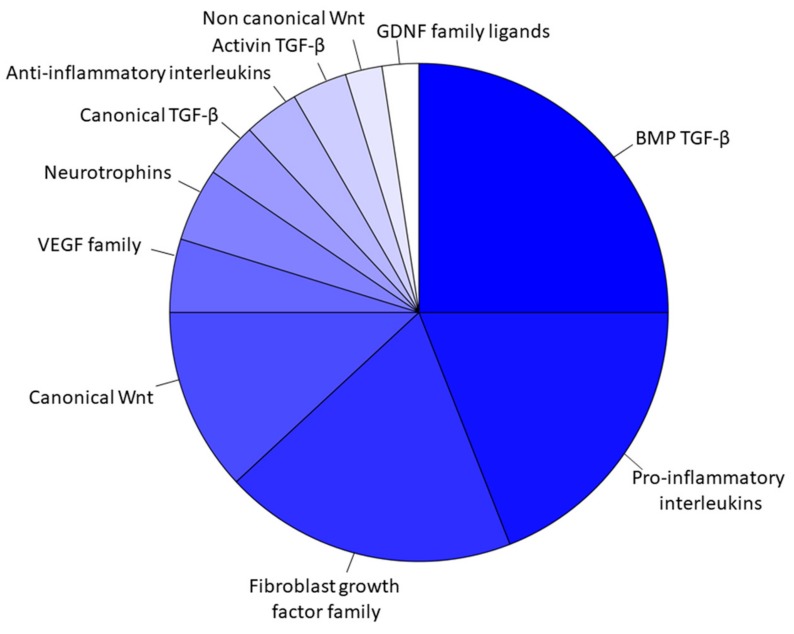
Genes representation of the EVs transcriptomic profile.

**Figure 4:**
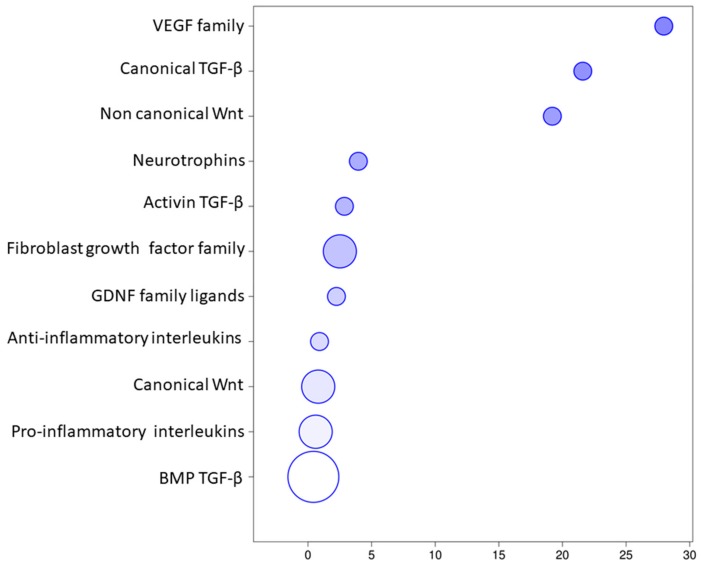
Genes distribution of the EVs transcriptomic profile. The size of the bubble indicate the amount of genes in the group while the x axis shows the median of the expression level of the genes in the group.

The transcriptomic analysis of the EVs revealed that 84 transcripts belonged to the “Receptor ligands” classification in HUGO website and specifically they can be divided in 7 groups including “Transforming growth factor β superfamily”, “Interleukins”, “Fibroblast growth factor family”, “Wnt family”,” VEGF family”, “Neurotrophins”, and “GDNF family ligands”. The classification of these transcripts in the groups in detail is represented in the Figure 3. Specifically, 19 of them belonged to the *Interleukins* (Table 1) that take part mainly in inflammatory and immune responses. Furthermore, 27 transcripts belonged to “TGF-β signaling” (Table 2) and it includes 3 transcripts of the canonical pathway, 21 of the bone morphogenic proteins involved in osteoblast differentiation and neurogenesis and 3 of the activin expressed in the initial development. They play a role in the ossification and bone mineralization, in neuron development and differentiation as well as negative regulation of neuron apoptotic process and in vasculogenesis and regulation of the angiogenesis. The “Wnt signaling” (Table 3) included 10 transcripts of the canonical pathway that regulates gene transcription and cell cycle but also 2 genes of the non-canonical calcium mediated pathway that is responsible for the regulation of intracellular calcium. They are involved in osteoblast differentiation and bone mineralization but especially in the nervous system development and maintenance. The Table 4 includes 16 genes of the “Fibroblast growth factor family”, 2 genes of “GDNF family ligands”, 4 genes of the “VEGF family” and 4 “Neurotrophins” involved in a wide amount of processes of the basic or neuronal, bone or vascular development. Furthermore, the Figure 4 represents the distribution of the genes inside the 7 aforementioned groups using a bubble chart. The size of the bubble indicates the number of genes in that particular group while the position in the x axis shows the median distribution of the genes expression in the group. Noteworthy, the “VEGF family”, the “Canonical TGF-β”, and the “Non canonical Wnt” groups include few genes that are highly expressed by median, whereas “BMP TGF-β” contains most of the genes, but less expressed.

## 4. Discussion

The hGMSCs are interesting as candidates for cell therapy in tissue repair and regeneration, both due to their ability to differentiate in multiple lineage cells and through the secretion of EVs [28]. EVs released by hGMSCs are involved in a multitude of physiological events as important mediators of intercellular communication. Indeed, EVs act as vehicles to transfer lipids, proteins, and nucleic acids, such as mRNAs, microRNAs, and long non-coding RNA, in the receiving cells. In particular, it was demonstrated that EVs can mediate the horizontal mRNA transfer to a recipient cell, that in this way was able to produce the relative protein [29]. In this way, EVs are able to modify the phenotype and function of the receiving cells, modulating multiple cellular pathways and activating regenerative mechanisms. Therefore, the application of hGMSCs-derived EVs can provide a new strategy for tissue engineering and regenerative medicine [30].

Our research group has already characterized the hGMSCs-derived EVs, indicating that they represent a heterogeneous population of vesicles. In particular, two main dimensional populations were identified, where the average diameters of the 2 populations were 93 ± 24 nm and 1200 ± 400 nm, while the ζ-potential was −10.7 ± 0.9 mV. The atomic force microscope evidenced that EVs showed a central depression and a relatively smooth surface. In addition, hGMSCs-derived EVs showed the presence of specific membrane-associated proteins, such as CD9, CD63, CD81, and tumor suppressor gene 101 [31].

The analysis of our transcriptome revealed the presence of different transcripts belonging to several classes, such as nucleic acid binding, hydrolase, enzyme modulator, transcription factor. Moreover, our results showed that hGMSCs-derived EVs also secrete several ncRNAs, that may mediate self-renewal, differentiation, maturation, efficiency of cellular reprogramming and cell fate determination [32,33,34]. The ncRNAs are non-protein coding RNAs, which represent part of the genome that does not encode genetic information into proteins. They are broadly categorized into short ncRNAs and long ncRNAs (lncRNAs) or long intergenic ncRNA (lincRNA). These RNAs appear to comprise a hidden layer of internal signals that control various levels of gene expression in physiology and development, including chromatin architecture/epigenetic memory, transcription, RNA splicing, editing, translation and turnover [35]. Furthermore, the ncRNAs are known to be sorted into EVs thus modulating cellular processes [36].

In particular, our findings demonstrate that the most represented ncRNA classes were “Antisense RNAs” and “lncRNAs”. lncRNAs have now been recognized to represent an important class of transcripts in all organisms, with increasing impact along the evolutionary ladder. However, the mechanisms underlying EVs-associated lncRNAs secretion and their biological roles are less described and are only more recently starting to be explored [37]. Since ncRNAs are central to gene regulation and cellular fates, it can be speculated that most of the EVs-mediated regulatory roles elicited in cells/organs are mediated through ncRNAs. Therefore, EVs-derived ncRNAs are potential mediators of the paracrine effects of stem cells. In our study, the analysis of the transcriptome of the hGMSCs-derived EVs identified the presence of transcripts (*TGFB1*, *TGFB2*, *TGFB3*) that encoded respectively for the three Transforming Growth Factors-β (TGF-β) isoforms. All three TGF-β isoforms are involved in several functions in a variety of cell types, including regulation of proliferation, differentiation, wound healing, development and cytokine secretion [38,39]. Also, TGF-β1 is a pleiotropic growth factor with significant anti-inflammatory and immunosuppressive properties and plays a central roles in homeostasis of the immune system [40]. The results of our transcriptome are in accordance with the previous study of our research group that highlighted the therapeutic effects of TGF-β present in EVs derived from human Periodontal Ligament Stem Cells (hPDLSCs), in Experimental Autoimmune Encephalomyelitis (EAE), a mouse model of multiple sclerosis. The presence of TGF-β in hPDLSCs-derived EVs revealed through western blot analysis, suggested that the anti-inflammatory effect of EVs in EAE might be due to TGF-β [41]. The anti-inflammatory actions of TGF-β contained in CM derived from hGMSCs was evaluated in an in vivo study, conducted by Rajan et al. in mechanically injured murine motor-neuron-like NSC-34 cells. Specifically, western blot analysis results showed that, due to activating trophic factors expression such as TGF-β, CM derived from hGMSCs provided neuroprotection in scratch-injured motor-neuron-like NSC-34 cells by suppressing apoptosis, oxidative stress, and inflammation [42]. In line with our results, the presence for the mRNA encoding members of the TGF-β family was evidenced also in EVs derived from different MSCs. Specifically, EVs from porcine Adipose Tissue derived MSCs were particularly enriched for TGF-β related genes, containing high levels of several mRNAs that encode for protein ligands within the TGF-β family, including *TGFB1* and *TGFB3* [43]. *TGFB1* mRNA was also detected in Bone Marrow MSCs-derived EVs and in Cord Blood MSCs-derived EVs [29].

Our analysis also showed the presence of anti-inflammatory interleukins such as IL-37, IL-19, and IL-27. Their expression highlights the beneficial effects of the hGMSCs-derived EV in the role of inflammation disorders. Our transcriptomic analysis of hGMSCs-derived EVs showed the presence of 12 transcripts (*BMP2*, *BMP4*, *BMP7*, *BMP3*, *BMP8A*, *BMP8B*, *BMP6*, *BMP1*, *BMP5*, *BMP10*, *BMP15,* and *AMH*) that encode for the family of the Bone Morphogenetic Proteins (BMPs). All these transcripts, except *AMH*, *BMP10,* and *BMP15*, induce endochondral/intramembranous ossification and chondrogenesis. Indeed, inducing the differentiation of mesenchymal stem cells towards the osteoblastic lineage, they are fundamental for maintaining skeletal integrity and bone repair [44,45,46,47,48]. Additionally, in our transcriptome are present Growth Differentiation Factors (GDFs) (*GDF2*, *GDF3*, *GDF5*, *GDF6*, *GDF10,* and *GDF11*), secreted signaling molecules within the BMP family, that could be involved in bone and cartilage formation [44]. Diomede et al. showed that EVs derived from both hGMSCs and hPDLSCs, seeded on different scaffolds, in vitro, are able to promote the osteogenic differentiation of both hGMSCs and hPDLSCs. Specifically, EVs or polyethylenimine (PEI)-engineered EVs (PEI-EVs), in the presence of scaffolds, increased the osteogenic differentiation of hGMSCs and hPDLSCs as demonstrated by the increased gene expression of osteogenic markers, such as RUNX2 and BMP2/4 [49,50]. In detail, the increased gene expression of BMP2/4, found in this work is in accordance with our transcriptomic analysis and highlights the osteogenetic capacity of EVs. Also in vivo, the constructs enriched with EVs/PEI-EVs increased the levels of BMP2/4 in rats subjected to calvaria defects compared to a scaffold or oral hMSCs alone, thus obtaining the complete repair of the calvarial defect [49]. Therefore the osteogenetic capacity of EVs, already demonstrated in the previous studies [31,49], could be related to pro-osteogenic factors that they contain, as showed in this work.

Interestingly, the analysis of the hGMSCs-derived EVs reveals 4 members of Wnt family (*WNT4*, *WNT11*, *WNT10B,* and *WNT16*) that encode to Wnt family members. Specifically, they are involved in bone tissue development by the promotion of the osteoblast differentiation and activation and as well as the inhibition of osteoblast apoptosis [50,51]. Thus, the presence of these transcripts in our hGMSCs-derived EVs highlights their importance in the field of bone regeneration.

Additionally, in our transcriptome, we found *INHA* and *INHBA* that are implicated in the osteogenesis process. *INHA* encodes for the α subunit of the inhibins whereas *INHBA* encodes for the subunit β. Inhibins are involved in the regulation of different functions such as hypothalamic and pituitary hormone secretion, gonadal hormone secretion, germ cell development and maturation, erythroid differentiation, insulin secretion, nerve cell survival, embryonic axial development, or bone growth, depending on their subunit composition. In particular, it has been already shown that the overexpression of the Inhibin A increases bone formation stimulating mature osteoblast activity [52]. Therefore, the presence of this transcript in our hGMSCs-derived EVs could demonstrate the possible therapeutic implication of EVs in bone remodeling.

Furthermore, our analysis also identified the presence of 4 proteins belonging to the Vascular Endothelial Growth Factor (VEGF) family, including VEGF-A, VEGF-C, VEGF-B, and Placental Growth Factor (PGF). All these proteins are crucial regulators of vascular development during embryogenesis as well as blood-vessel formation in the adult [53]. Specifically, VEGF-A protein, commonly called VEGF, was the first member to be discovered and plays a key role in angiogenesis but is also important for bone growth and regeneration [54]. In rats subjected to calvarial defects, the scaffolds enriched with EVs/PEI-EVs and hPDLSCs improved also the vascularization process. Indeed, the pro-angiogenic factor VEGF resulted more expressed in the calvaria of the rats grafted with the scaffold [55]. Therefore, the expression of the pro-angiogenic factors in our EVs, as demonstrated in this work, supports the angiogenetic properties of EVs and their possible therapeutic role in the field of tissue repair. In compliance with our results, Ragni E. et al. showed that VEGFA mRNA was abundant in EVs from Bone Morrow MSCs and Cord Blood MSCs [29]. A proteomic study, showed that also EVs isolated from porcine adipose tissue-derived MSCs are enriched in VEGF. Moreover, it has also been demonstrated that EVs may not only deliver the VEGF protein but also upregulate its production in recipient cells [56].

The transcriptomic analysis highlighted also the presence of growth factors (*FGF1*, *FGF2*, *FGF6*, *FGF9*, *FGF18*, *FGF23*, *FGF10*, *FGF5*, *FGF13*, *FGF19*, *FGF12*, *FGF7*, *FGF11*, *FGF20*, *FGF4,* and *FGF14*) belonging to the Fibroblast Growth Factor (FGF) family. FGFs are involved in many biological processes, including angiogenesis, embryogenesis, differentiation, and proliferation [57]. Specifically, *FGF1* and *FGF2* are potent angiogenic factors that induce the promotion of endothelial cell proliferation and the physical organization of endothelial cells into tube-like structures [58]. Additionally, they are also involved in the expression of osteogenic markers and mineralization, demonstrating a possible role in bone regeneration [59]. In line with our results, a mRNA transcriptomic analysis showed that *FGF2* and *FGF7* were consistently accumulated in Bone Marrow MSCs-derived EVs and in Cord Blood MSCs-derived EVs. In particular, *FGF7* was among most enriched transcripts in both types of EVs [29]. Moreover, exosomes derived from adipose MSCs were reported to contain *FGF1* transcript [60]. Interestingly, other members of this family, such as *FGF6*, *FGF9*, *FGF18,* and *FGF23*, show regulatory activity in bone development [61,62]. The expression of the FGFs in the transcriptomic profile of the hGMSCs-derived EVs support their potential for application in regenerative medicine. In addition, the FGFs play also a role in the nervous system development and maintenance [63]. Specifically, *FGF20*, *FGF14*, *FGF13,* and *FGF12* are involved in the regulation of synaptic plasticity and postsynaptic membrane potential, in the differentiation of the dopaminergic neurons and in regulation of the neuronal action potential as well as in learning and memory.

Also, Wnt family is involved in the homeostasis of neuron cells, from neurogenesis to neuron survival [64,65]. In detail, *WNT10A* takes place in neural crest cell differentiation, *WNT3* in the regulation of neurogenesis [66], *WNT2B* in forebrain reorganization while *WNT8A*, *WNT9A,* and *WNT5B* are involved in aspecific neuron differentiation [66,67]. On the other hand, *WNT5A* and *WNT7A* are the best characterized; they regulate the assembly of the excitatory and inhibitory synapsis, the neurotransmitter secretion, the neuron projection development and the ion concentration in post-synaptic neurons [67,68].

The regulation of the nervous system is mediated also by neurotrophins, neurotrophic factor specialized in the promotion of neuron survival, escaping from programmed neural cell death and stimulating the neurogenesis. Additionally, they can regulate axonal growth in neurons [69]. Interestingly, the transcriptomic profile of the hGMSCs-derived EVs reveals *NGF*, *BDNF*, *NTF3,* and *NTF4* that respectively encode for the neurotrophins Nerve Growth Factor (*NGF*), Brain Derived Neurotrophic Factor (*BDNF*), Neurotrophin 3 (*NTF3*), and Neurotrophin 4 (*NTF4*). They are specifically implied in memory, nerve development, axonogenesis and axon guidance, neuronal survival, proliferation, and negative regulation of apoptosis [70]. *NGF*, the best characterized neurotrophin, is mainly involved in the intra-cellular signal communication from the axon to the cell body. *BDNF* is a neurotrophic factor known to promote neural cell proliferation and survival in the developing human brain [71,72]. *BDNF* protein expression is especially high in the hippocampus, but it can also affect the survival and proliferation rate of several neural cells, including the cerebellar and cortical neurons [71,73]. An in vitro study of scratch-injured NSC-34 cells treated with hGMSCs-derived CM, the neuroprotective actions of *BDNF* and *NTF3* was evaluated. More than untreated injured cells, scratch-injured NSC-34 cells treated with hGMSCs-derived CM showed an increased presence of neurotrophins *BDNF* and *NTF3* [42]. In compliance with our results, this data demonstrated how hGMSCs-derived CM or EVs may provide neuroprotection via elevating the level of *BDNF* and *NTF3*. In addition, hGMSCs-derived EVs profile shows *GDNF* and *PSPN* transcripts that are neurotrophic factors of the Glial cell line-derived neurotrophic factor family. Their role in nervous system development and axon guidance in dopaminergic and motoneurons, as well as in neuronal survival, differentiation and plasticity, find an implication in neurodegenerative disease like Parkinson’s disease and amyotrophic lateral sclerosis [74]. Also exosomes derived from adipose derived MSCs were reported to contain *GDNF*, *BDNF,* and *NGF* transcripts, that may play a role in the nerve regeneration [60]. *NGF* and *BDNF* transcripts were also present in Bone Marrow MSCs-derived EVs and in Cord Blood MSCs-derived EVs [29].

A deep characterization of the transcripts identified in the transcriptome would be important and, in this context, it would be interesting to evaluate their role and how their lack can affect the vesicular function or cell fate. Several studies evaluated the role of specific transcripts knocking down the relative gene in order to evaluate the most important functions. In particular, a study demonstrated that the knockdown of Wnt4 in human umbilical cord derived MSCs exosomes inhibited the activation of β-catenin and skin cell proliferation and migration, reducing the therapeutic effects in vivo. These data indicated that Wnt4 is the main mediator carried by in human umbilical cord derived MSCs exosomes for wound healing [75]. Moreover, another work demonstrated that *VEGF* mRNA and protein contained inside EVs is the responsible of the protection in hyperoxic lung injury, indeed VEGF knockout EVs were not able to mediate the protective effects [76]. These data may indicate that several transcripts may be necessary for EVs to exert a specific function. However, it is important to notice that EVs’ content is not static and it can change in response to different cell stimuli, such as differentiation [77,78]. Sun et al. evidenced that different miRNA were differentially expressed with and without chondrogenic induction [77]. In addition, their content may vary also during the different stage of osteogenic differentiation, as demonstrated by Wang et al., that evidenced that the EVs content of miRNA was different between early and late stages of differentiation and in particular the cargo transferred during the late stage of differentiation induced osteogenic differentiation [78].

## 5. Conclusions

The transcriptomic profile of hGMSCs-derived EVs evidenced that the transcripts belonged to different protein classes, but also ncRNAs, with important functions in the regulation of gene expression, were present. Our analysis reveals transcripts that encode for proteins of the Interleukins, TGF-β, BMPs, GDFs, Wnt, VEGF, FGF, and neurotrophins families. They are specifically involved in inflammatory and immune response (interleukins), ossification and bone mineralization, in neuron development and differentiation, as well as negative regulation of neuron apoptotic process and in vasculogenesis and regulation of angiogenesis (TGF-β, BMPs, and GDFs). Furthermore, osteoblast differentiation and bone mineralization, especially in nervous system development and maintenance, involves Wnt, and a huge amount of processes—basic or neuronal, bone or vascular development—involve VEGF, FGF, and neurotrophins. Therefore, our results show that hGMSCs-derived EVs could effectively support therapeutic approaches in the field of regenerative medicine.

## Figures and Tables

**Table 1:** EVs transcripts encoding for *Interleukins.*

Transcript	Name	Biological Process
*IL19*	Interleukin 19	Apoptotic process; immune response
*IL37*	Interleukin 37	Inflammatory response; immune response; neutrophil chemotaxis
*IL21*	Interleukin 21	Immune response; positive regulation of cell population proliferation; positive regulation of B cell proliferation; positive regulation of tissue remodeling; positive regulation of T cell proliferation
*IL17A*	Interleukin 17A	Inflammatory response; immune response; positive regulation of osteoclast differentiation; apoptotic process; fibroblast activation
*IL15*	Interleukin 15	Neutrophil activation; positive regulation of cell population proliferation; lymph node development; inflammatory response; immune response; positive regulation of tissue remodeling; macrophage differentiation; cell-cell signaling; cell maturation; cellular response to vitamin D
*IL12A*	Interleukin 12A	Cell cycle arrest; positive regulation of natural killer cell mediated cytotoxicity directed against tumor cell target; immune response; positive regulation of dendritic cell chemotaxis; defense response to Gram-positive bacterium
*IL12B*	Interleukin 12B	Positive regulation of lymphocyte proliferation; positive regulation of tissue remodeling; sensory perception of pain; positive regulation of osteoclast differentiation; defense response to Gram-negative bacterium; positive regulation of memory T cell differentiation
*IL6*	Interleukin 6	Regulation of dendritic cell differentiation; positive regulation of osteoblast differentiation; regulation of odontoblast differentiation; negative regulation of neuron apoptotic process; inflammatory response; liver regeneration; immune response; regulation of osteoclast differentiation; positive regulation of biomineral tissue development; neuron differentiation
*IL7*	Interleukin 7	Cell-cell signaling; positive regulation of organ growth; immune response; bone resorption; negative regulation of apoptotic process; positive regulation of T cell differentiation; positive regulation of B cell proliferation
*IL5*	Interleukin 5	Positive regulation of eosinophil differentiation; inflammatory response; positive regulation of B cell proliferation; positive regulation of podosome assembly
*IL25*	Interleukin 25	Inflammatory response to antigenic stimulus; eosinophil differentiation
*IL24*	Interleukin 24	Positive regulation of cell population proliferation
*IL27*	Interleukin 27	Inflammatory response; response to bacterium; innate immune response; regulation of T cell proliferation
*IL32*	Interleukin 32	Immune response; cell adhesion
*IL1B*	Interleukin 1 β	Activation of MAPK activity; positive regulation of T cell mediated immunity; inflammatory response, apoptotic process; cell-cell signaling; positive regulation of vascular endothelial growth factor production; astrocyte activation; positive regulation of glial cell proliferation
*IL36B*	Interleukin 36 β	Inflammatory response; innate immune response; neutrophil chemotaxis
*IL16*	Interleukin 16	Immune response; leukocyte chemotaxis
*IL36G*	Interleukin 36 γ	Inflammatory response; innate immune response; cell-cell signaling; neutrophil chemotaxis
*IL33*	Interleukin 33	Microglial cell activation involved in immune response; microglial cell proliferation; negative regulation of interferon-γ production

EVs transcripts encoding for Interleukins. The column Name highlights the gene approved names retrieved by “*HUGO Gene Nomenclature Committee*” website. Panther database provides a set of biological processes in which the transcripts are involved.

**Table 2:** EVs transcripts encoding for protein of the *TGF-β* family.

Transcript	Name	Biological Process
*TGFB1*	Transforming Growth Factor β 1	Hematopoietic progenitor cell differentiation; embryonic liver development; positive regulation of protein import into nucleus; positive regulation of vascular permeability; epithelial to mesenchymal transition; positive regulation of MAP kinase activity; positive regulation of cell population proliferation; lymph node development; heart development; positive regulation of cell division; neural tube development; BMP signaling pathway; positive regulation of bone mineralization; regulation of blood vessel remodeling; vasculogenesis; positive regulation of microglia differentiation
*TGFB2*	Transforming Growth Factor β 2	Negative regulation of angiogenesis; glial cell migration; kidney development; positive regulation of ossification; epithelial to mesenchymal transition; heart development; cranial skeletal system development; neuron development; BMP signaling pathway; cardiac muscle proliferation; generation of neurons
*TGFB3*	Transforming Growth Factor β 3	Digestive tract development; BMP signaling pathway; positive regulation of bone mineralization; negative regulation of neuron apoptotic process; positive regulation of epithelial to mesenchymal transition; ossification involved in bone remodeling
*BMP7*	Bone Morphogenetic Protein 7	Axon guidance; skeletal system development; ossification; hindbrain development; cellular response to BMP stimulus; positive regulation of bone mineralization; response to vitamin D; neuron projection morphogenesis; cardiac muscle tissue development; positive regulation of dendrite development
*AMH*	Anti-Mullerian Hormone	Cell-cell signaling; BMP signaling pathway
*GDF9*	Growth Differentiation Factor 9	Positive regulation of cell population proliferation; BMP signaling pathway; negative regulation of cell growth
*BMP4*	Bone Morphogenetic Protein 4	Positive regulation of kidney development; cellular response to BMP stimulus; telencephalon development; positive regulation of neuron differentiation; positive regulation of osteoblast differentiation; BMP signaling pathway involved in heart induction
*BMP3*	Bone Morphogenetic Protein 3	Regulation of MAPK cascade; skeletal system development; osteoblast differentiation
*BMP8A*	Bone Morphogenetic Protein 8a	Regulation of MAPK cascade, ossification
*BMP8B*	Bone Morphogenetic Protein 8b	Regulation of MAPK cascade; skeletal system development; ossification
*BMP6*	Bone Morphogenetic Protein 6	Positive regulation of neuron differentiation; positive regulation of osteoblast differentiation; skeletal system development; kidney development; cellular response to BMP stimulus; positive regulation of bone mineralization
*GDF3*	Growth Differentiation Factor 3	Negative regulation of BMP signaling pathway; skeletal system development
*GDF2*	Growth Differentiation Factor 2	Positive regulation of angiogenesis; blood vessel morphogenesis; ossification; osteoblast differentiation
*GDF1*	Growth Differentiation Factor 1	Regulation of MAPK cascade; regulation of apoptotic process; BMP signaling pathway
*GDF6*	Growth Differentiation Factor 6	Positive regulation of neuron differentiation; regulation of MAPK cascade
*GDF5*	Growth Differentiation Factor 5	Negative regulation of mesenchymal apoptotic process; positive regulation of neuron differentiation; ossification involved in bone remodeling; negative regulation of neuron apoptotic process
*BMP2*	Bone Morphogenetic Protein 2	Activation of MAPK activity; inflammatory response; skeletal system development; positive regulation of osteoblast proliferation; bone mineralization involved in bone maturation; telencephalon development; positive regulation of neuron differentiation; positive regulation of osteoblast differentiation; heart development
*BMP1*	Bone Morphogenetic Protein 1	Skeletal system development; ossification
*BMP10*	Bone Morphogenetic Protein 10	Positive regulation of cardiac muscle cell proliferation; regulation of MAPK cascade; positive regulation of cell proliferation involved in heart morphogenesis
*BMP5*	Bone Morphogenetic Protein 5	Skeletal system development; positive regulation of dendritic development
*BMP15*	Bone Morphogenetic Protein 15	Regulation of MAPK cascade; BMP signaling pathway
*GDF15*	Growth Differentiation Factor 15	Positive regulation of MAPK cascade; activation of MAPK cascade; BMP signaling pathway
*GDF11*	Growth Differentiation Factor 11	Regulation of MAPK cascade; skeletal system development
*GDF10*	Growth Differentiation Factor 10	Regulation of MAPK cascade; skeletal system development; BMP signaling pathway
*INHA*	Inhibin Subunit α	Regulation of MAPK cascade; skeletal system development; negative regulation of interferon-γ biosynthetic process
*INHBA*	Inhibin Subunit β A	Regulation of MAPK cascade; nervous system development; negative regulation of interferon-γ biosynthetic process; GABAergic neuron differentiation
*INHBC*	Inhibin Subunit β C	Regulation of MAPK cascade

EVs transcripts encoding for Interleukins. The column Name highlights the gene approved names retrieved by “*HUGO Gene Nomenclature Committee*” website. Panther database provides a set of biological processes in which the transcripts are involved.

**Table 3:** EVs transcripts encoding for protein of the *Wnt* family.

Transcript	Name	Biological Process
*WNT2B*	Wnt Family Member 2B	Neuron differentiation; forebrain reorganization; canonical Wnt signaling
*WNT3*	Wnt Family Member 3	Axon guidance; canonical Wnt signaling pathway involved in osteoblast differentiation; pathway involved in midbrain dopaminergic neuron differentiation; neuron differentiation; regulation of neurogenesis
*WNT10A*	Wnt Family Member 10A	Neural crest cell differentiation; neuron differentiation; canonical Wnt signaling pathway
*WNT10B*	Wnt Family Member 10B	Positive regulation of osteoblast differentiation; regulation of skeletal muscle tissue development; canonical Wnt signaling pathway; positive regulation of bone mineralization; neuron differentiation
*WNT4*	Wnt Family Member 4	Kidney development; positive regulation of bone mineralization; neuron differentiation; canonical Wnt signaling pathway
*WNT8A*	Wnt Family Member 8A	Neuron differentiation; canonical Wnt signaling
*WNT9A*	Wnt Family Member 9A	Neuron differentiation; canonical Wnt signaling
*WNT16*	Wnt Family Member 16	Bone remodeling; neuron differentiation
*WNT7A*	Wnt Family Member 7A	Central nervous system vasculogenesis; axonogenesis; positive regulation of synapse assembly; neuron differentiation; excitatory synapse assembly; neurotransmitter secretion; dendritic spine morphogenesis; regulation of axon diameter; cell proliferation in forebrain
*WNT11*	Wnt Family Member 11	Neuron differentiation; artery morphogenesis; osteoblast differentiation; bone mineralization
*WNT5B*	Wnt Family Member 5B	Neuron differentiation
*WNT5A*	Wnt Family Member 5A	Axon guidance; positive regulation of angiogenesis; activation of MAPK activity; inhibitory synapse assembly; excitatory synapse assembly; positive regulation of ossification; chemoattraction of serotonergic neuron axon; chemorepulsion of dopaminergic neuron axon; positive regulation of fibroblast proliferation; positive regulation of protein localization to synapse; positive regulation of neuron projection development; neuron differentiation; Wnt signaling pathway involved in midbrain dopaminergic neuron differentiation; regulation of postsynaptic cytosolic calcium ion concentration

EVs transcripts encoding for Interleukins. The column Name highlights the gene approved names retrieved by “*HUGO Gene Nomenclature Committee*” website. Panther database provides a set of biological processes in which the transcripts are involved.

**Table 4:** EVs transcripts encoding for *Growth factors*.

Transcript	Name	Biological Process
*FGF10*	Fibroblast Growth Factor 10	Activation of MAPK activity; angiogenesis; positive regulation of fibroblast proliferation; radial glial cell differentiation; positive regulation of MAPK cascade; pituitary gland development; actin cytoskeletal reorganization; tissue regeneration; blood vessel remodeling
*FGF5*	Fibroblast Growth Factor 5	Nervous system development; glial cell differentiation
*FGF2*	Fibroblast Growth Factor 2	Positive regulation of cardiac muscle cell proliferation; positive regulation of angiogenesis; activation of MAPK activity; nervous system development; negative regulation of cell death; somatic stem cell population maintenance;
*FGF13*	Fibroblast Growth Factor 13	Nervous system development; hippocampus development; learning; memory; cerebral cortex cell migration
*FGF19*	Fibroblast Growth Factor 19	MAPK cascade; nervous system development
*FGF18*	Fibroblast Growth Factor 18	Positive regulation of MAP kinase activity; positive regulation of angiogenesis
*FGF1*	Fibroblast Growth Factor 1	Positive regulation of MAP kinase activity; positive regulation of angiogenesis; lung development
*FGF9*	Fibroblast Growth Factor 9	Positive regulation of cardiac muscle cell proliferation; angiogenesis; osteoblast differentiation; substantia nigra development
*FGF12*	Fibroblast Growth Factor 12	Regulation of neuronal action potential; neuromuscular process; nervous system development; chemical synaptic transmission; heart development
*FGF7*	Fibroblast Growth Factor 7	Epidermis development; actin cytoskeleton reorganization
*FGF6*	Fibroblast Growth Factor 6	MAPK cascade; angiogenesis
*FGF11*	Fibroblast Growth Factor 11	Nervous system development
*FGF4*	Fibroblast Growth Factor 4	Stem cell population maintenance; MAPK cascade
*FGF23*	Fibroblast Growth Factor 23	Vitamin D catabolic process; negative regulation of osteoblast differentiation; negative regulation of bone mineralization
*FGF20*	Fibroblast Growth Factor 20	Positive regulation of dopaminergic neuron differentiation; negative regulation of neuron apoptotic process
*FGF14*	Fibroblast Growth Factor 14	Regulation of postsynaptic membrane potential; regulation of synaptic vesicle recycling; regulation of synaptic plasticity; nervous system development
*PSPN*	Persephin	Axon guidance; nervous system development
*GDNF*	Glial Cell Derived Neurotrophic Factor	Axon guidance; nervous system development
*PGF*	Placental Growth Factor	Positive regulation of angiogenesis
*VEGFA*	Vascular Endothelial Growth Factor A	Positive regulation of angiogenesis; response to hypoxia; positive regulation of MAP kinase activity; commissural neuron axon guidance; positive regulation of blood vessel endothelial cell migration; dopaminergic neuron differentiation; hearth morphogenesis; positive regulation of neuroblast differentiation; artery morphogenesis; positive regulation of axon extension involved in axon guidance
*VEGFC*	Vascular Endothelial Growth Factor C	Positive regulation of angiogenesis; response to hypoxia; positive regulation of neuroblast proliferation; negative regulation of blood pressure
*VEGFB*	Vascular Endothelial Growth Factor B	Positive regulation of angiogenesis; negative regulation of neuron apoptotic process; response to hypoxia
*NGF*	Nerve Growth Factor	Neuron projection morphogenesis; positive regulation of neuron differentiation; memory; nerve development; negative regulation of neuron apoptotic process; positive regulation of axonogenesis; peripheral nervous system development
*NTF3*	Neurotrophin 3	Nervous system development; neuron projection morphogenesis; regulation of neuron differentiation; memory; nerve development; negative regulation of neuron apoptotic process; peripheral nervous system development
*NTF4*	Neurotrophin 4	Neuron projection morphogenesis; peripheral nervous system development; regulation of neuron differentiation; nerve development; negative regulation of neuron apoptotic process; long-term memory
*BDNF*	Brain Derived Neurotrophic Factor	Nervous system development; neuron projection morphogenesis; axon guidance; regulation of neuron differentiation; memory; nerve development; negative regulation of neuron apoptotic process; positive regulation of neuron projection development

EVs transcripts encoding for Interleukins. The column Name highlights the gene approved names retrieved by “*HUGO Gene Nomenclature Committee*” website. Panther database provides a set of biological processes in which the transcripts are involved.

## Supplementary Materials

The following are available online at https://www.mdpi.com/2073-4425/11/2/118/s1, Table S1: List of transcripts included in the EVs analysis.

## References

[1] Sharpe P.T. (2016). Dental mesenchymal stem cells. Development.

[2] Du L., Yang P., Ge S. (2016). Isolation and characterization of human gingiva-derived mesenchymal stem cells using limiting dilution method. J. Dent. Sci..

[3] Gronthos S., Mankani M., Brahim J., Robey P.G., Shi S. (2000). Postnatal human dental pulp stem cells (dpscs) in vitro and in vivo. Proc. Natl. Acad. Sci. USA.

[4] Sonoyama W., Liu Y., Fang D., Yamaza T., Seo B.-M., Zhang C., Liu H., Gronthos S., Wang C.-Y., Shi S. (2006). Mesenchymal stem cell-mediated functional tooth regeneration in swine. PLoS ONE.

[5] Miura M., Gronthos S., Zhao M., Lu B., Fisher L.W., Robey P.G., Shi S. (2003). Shed: Stem cells from human exfoliated deciduous teeth. Proc. Natl. Acad. Sci. USA.

[6] Morsczeck C., Götz W., Schierholz J., Zeilhofer F., Kühn U., Möhl C., Sippel C., Hoffmann K. (2005). Isolation of precursor cells (pcs) from human dental follicle of wisdom teeth. Matrix Biol..

[7] Seo B.-M., Miura M., Gronthos S., Bartold P.M., Batouli S., Brahim J., Young M., Robey P.G., Wang C.Y., Shi S. (2004). Investigation of multipotent postnatal stem cells from human periodontal ligament. Lancet.

[8] Fournier B., Loison-Robert L., Ferre F., Owen G., Larjava H., Häkkinen L. (2016). Characterisation of human gingival neural crest-derived stem cells in monolayer and neurosphere cultures. Eur. Cell Mater..

[9] Xu X., Chen C., Akiyama K., Chai Y., Le A.D., Wang Z., Shi S. (2013). Gingivae contain neural-crest- and mesoderm-derived mesenchymal stem cells. J. Dent. Res..

[10] Diomede F., Zini N., Pizzicannella J., Merciaro I., Pizzicannella G., D’Orazio M., Piattelli A., Trubiani O. (2018). 5-aza exposure improves reprogramming process through embryoid body formation in human gingival stem cells. Front. Genet..

[11] Nuti N., Corallo C., Chan B., Ferrari M., Gerami-Naini B. (2016). Multipotent differentiation of human dental pulp stem cells: A literature review. Stem Cell Rev. Rep..

[12] Jin S.H., Lee J.E., Yun J.H., Kim I., Ko Y., Park J.B. (2015). Isolation and characterization of human mesenchymal stem cells from gingival connective tissue. J. Periodontal Res..

[13] Zhang Q.Z., Su W.R., Shi S.H., Wilder-Smith P., Xiang A.P., Wong A., Nguyen A.L., Kwon C.W., Le A.D. (2010). Human gingiva-derived mesenchymal stem cells elicit polarization of m2 macrophages and enhance cutaneous wound healing. Stem Cells.

[14] Fawzy El-Sayed K.M., Dörfer C.E. (2016). Gingival mesenchymal stem/progenitor cells: A unique tissue engineering gem. Stem Cells Int..

[15] Dominici M., Le Blanc K., Mueller I., Slaper-Cortenbach I., Marini F., Krause D., Deans R., Keating A., Prockop D., Horwitz E. (2006). Minimal criteria for defining multipotent mesenchymal stromal cells. The international society for cellular therapy position statement. Cytotherapy.

[16] Gugliandolo A., Diomede F., Cardelli P., Bramanti A., Scionti D., Bramanti P., Trubiani O., Mazzon E. (2018). Transcriptomic analysis of gingival mesenchymal stem cells cultured on 3 d bioprinted scaffold: A promising strategy for neuroregeneration. J. Biomed. Mater. Res. Part A.

[17] Gugliandolo A., Diomede F., Scionti D., Bramanti P., Trubiani O., Mazzon E. (2019). The role of hypoxia on the neuronal differentiation of gingival mesenchymal stem cells: A transcriptional study. Cell Transplant..

[18] Mao Q., Nguyen P.D., Shanti R.M., Shi S., Shakoori P., Zhang Q., Le A.D. (2019). Gingiva-derived mesenchymal stem cell-extracellular vesicles activate schwann cell repair phenotype and promote nerve regeneration. Tissue Eng. Part A.

[19] Zhang P., Yeo J.C., Lim C.T. (2019). Advances in technologies for purification and enrichment of extracellular vesicles. SLAS Technol. Transl. Life Sci. Innov..

[20] Camussi G., Deregibus M.-C., Bruno S., Grange C., Fonsato V., Tetta C. (2011). Exosome/microvesicle-mediated epigenetic reprogramming of cells. Am. J. Cancer Res..

[21] Valadi H., Ekström K., Bossios A., Sjöstrand M., Lee J.J., Lötvall J.O. (2007). Exosome-mediated transfer of mrnas and micrornas is a novel mechanism of genetic exchange between cells. Nat. Cell Biol..

[22] del Conde I., Shrimpton C.N., Thiagarajan P., López J.A. (2005). Tissue-factor–bearing microvesicles arise from lipid rafts and fuse with activated platelets to initiate coagulation. Blood.

[23] Diomede F., Gugliandolo A., Scionti D., Merciaro I., Cavalcanti M., Mazzon E., Trubiani O. (2018). Biotherapeutic effect of gingival stem cells conditioned medium in bone tissue restoration. Int. J. Mol. Sci..

[24] Bolger A.M., Lohse M., Usadel B. (2014). Trimmomatic: A flexible trimmer for illumina sequence data. Bioinformatics.

[25] Dobin A., Davis C.A., Schlesinger F., Drenkow J., Zaleski C., Jha S., Batut P., Chaisson M., Gingeras T.R. (2013). Star: Ultrafast universal rna-seq aligner. Bioinformatics.

[26] Trapnell C., Hendrickson D.G., Sauvageau M., Goff L., Rinn J.L., Pachter L. (2013). Differential analysis of gene regulation at transcript resolution with rna-seq. Nat. Biotechnol..

[27] Mi H., Muruganujan A., Ebert D., Huang X., Thomas P.D. (2019). Panther version 14: More genomes, a new panther go-slim and improvements in enrichment analysis tools. Nucleic Acids Res..

[28] Xin H., Li Y., Chopp M. (2014). Exosomes/mirnas as mediating cell-based therapy of stroke. Front. Cell. Neurosci..

[29] Ragni E., Banfi F., Barilani M., Cherubini A., Parazzi V., Larghi P., Dolo V., Bollati V., Lazzari L. (2017). Extracellular vesicle-shuttled mrna in mesenchymal stem cell communication. Stem Cells.

[30] Ratajczak J., Wysoczynski M., Hayek F., Janowska-Wieczorek A., Ratajczak M. (2006). Membrane-derived microvesicles: Important and underappreciated mediators of cell-to-cell communication. Leukemia.

[31] Diomede F., Gugliandolo A., Cardelli P., Merciaro I., Ettorre V., Traini T., Bedini R., Scionti D., Bramanti A., Nanci A. (2018). Three-dimensional printed pla scaffold and human gingival stem cell-derived extracellular vesicles: A new tool for bone defect repair. Stem Cell Res. Ther..

[32] Judson R.L., Babiarz J.E., Venere M., Blelloch R. (2009). Embryonic stem cell-specific micrornas promote induced pluripotency. Nat. Biotechnol..

[33] Zhang W., Dong R., Diao S., Du J., Fan Z., Wang F. (2017). Differential long noncoding rna/mrna expression profiling and functional network analysis during osteogenic differentiation of human bone marrow mesenchymal stem cells. Stem Cell Res. Ther..

[34] Nawaz M., Fatima F., Vallabhaneni K.C., Penfornis P., Valadi H., Ekstrom K., Kholia S., Whitt J.D., Fernandes J.D., Pochampally R. (2016). Extracellular vesicles: Evolving factors in stem cell biology. Stem Cells Int..

[35] Mattick J.S., Makunin I.V. (2006). Non-coding rna. Hum. Mol. Genet..

[36] Fatima F., Ekstrom K., Nazarenko I., Maugeri M., Valadi H., Hill A.F., Camussi G., Nawaz M. (2017). Non-coding rnas in mesenchymal stem cell-derived extracellular vesicles: Deciphering regulatory roles in stem cell potency, inflammatory resolve, and tissue regeneration. Front. Genet..

[37] Fatima F., Nawaz M. (2017). Vesiculated long non-coding rnas: Offshore packages deciphering trans-regulation between cells, cancer progression and resistance to therapies. Non-Coding RNA.

[38] Goumans M.J., Valdimarsdottir G., Itoh S., Rosendahl A., Sideras P., ten Dijke P. (2002). Balancing the activation state of the endothelium via two distinct tgf-β type i receptors. EMBO J..

[39] Jian H., Shen X., Liu I., Semenov M., He X., Wang X.F. (2006). Smad3-dependent nuclear translocation of β-catenin is required for tgf-beta1-induced proliferation of bone marrow-derived adult human mesenchymal stem cells. Genes Dev..

[40] Li M.O., Flavell R.A. (2008). Contextual regulation of inflammation: A duet by transforming growth factor-β and interleukin-10. Immunity.

[41] Rajan T.S., Giacoppo S., Diomede F., Ballerini P., Paolantonio M., Marchisio M., Piattelli A., Bramanti P., Mazzon E., Trubiani O. (2016). The secretome of periodontal ligament stem cells from ms patients protects against eae. Sci. Rep..

[42] Rajan T.S., Diomede F., Bramanti P., Trubiani O., Mazzon E. (2017). Conditioned medium from human gingival mesenchymal stem cells protects motor-neuron-like nsc-34 cells against scratch-injury-induced cell death. Int. J. Immunopathol. Pharmacol..

[43] Eirin A., Riester S.M., Zhu X.Y., Tang H., Evans J.M., O’Brien D., van Wijnen A.J., Lerman L.O. (2014). Microrna and mrna cargo of extracellular vesicles from porcine adipose tissue-derived mesenchymal stem cells. Gene.

[44] Carreira A.C., Alves G.G., Zambuzzi W.F., Sogayar M.C., Granjeiro J.M. (2014). Bone morphogenetic proteins: Structure, biological function and therapeutic applications. Arch. Biochem. Biophys..

[45] Majumdar M.K., Wang E., Morris E.A. (2001). Bmp-2 and bmp-9 promotes chondrogenic differentiation of human multipotential mesenchymal cells and overcomes the inhibitory effect of il-1. J. Cell. Physiol..

[46] Ye M., Berry-Wynne K.M., Asai-Coakwell M., Sundaresan P., Footz T., French C.R., Abitbol M., Fleisch V.C., Corbett N., Allison W.T. (2009). Mutation of the bone morphogenetic protein gdf3 causes ocular and skeletal anomalies. Hum. Mol. Genet..

[47] McPherron A.C., Lawler A.M., Lee S.-J. (1999). Regulation of anterior/posterior patterning of the axial skeleton by growth/differentiation factor 11. Nat. Genet..

[48] Wang J., Yu T., Wang Z., Ohte S., Yao R.E., Zheng Z., Geng J., Cai H., Ge Y., Li Y. (2016). A new subtype of multiple synostoses syndrome is caused by a mutation in gdf6 that decreases its sensitivity to noggin and enhances its potency as a bmp signal. J. Bone Miner. Res..

[49] Diomede F., D’aurora M., Gugliandolo A., Merciaro I., Ettorre V., Bramanti A., Piattelli A., Gatta V., Mazzon E., Fontana A. (2018). A novel role in skeletal segment regeneration of extracellular vesicles released from periodontal-ligament stem cells. Int. J. Nanomed..

[50] Westendorf J.J., Kahler R.A., Schroeder T.M. (2004). Wnt signaling in osteoblasts and bone diseases. Gene.

[51] Jing H., Su X., Gao B., Shuai Y., Chen J., Deng Z., Liao L., Jin Y. (2018). Epigenetic inhibition of wnt pathway suppresses osteogenic differentiation of bmscs during osteoporosis. Cell Death Dis..

[52] Perrien D.S., Akel N.S., Edwards P.K., Carver A.A., Bendre M.S., Swain F.L., Skinner R.A., Hogue W.R., Nicks K.M., Pierson T.M. (2007). Inhibin a is an endocrine stimulator of bone mass and strength. Endocrinology.

[53] Olsson A.K., Dimberg A., Kreuger J., Claesson-Welsh L. (2006). Vegf receptor signaling—In control of vascular function. Nat. Rev. Mol. Cell Biol..

[54] Grosso A., Burger M.G., Lunger A., Schaefer D.J., Banfi A., Di Maggio N. (2017). It takes two to tango: Coupling of angiogenesis and osteogenesis for bone regeneration. Front. Bioeng. Biotechnol..

[55] Pizzicannella J., Gugliandolo A., Orsini T., Fontana A., Ventrella A., Mazzon E., Bramanti P., Diomede F., Trubiani O. (2019). Engineered extracellular vesicles from human periodontal-ligament stem cells increase vegf/vegfr2 expression during bone regeneration. Front. Physiol..

[56] Eirin A., Zhu X.Y., Puranik A.S., Woollard J.R., Tang H., Dasari S., Lerman A., van Wijnen A.J., Lerman L.O. (2016). Comparative proteomic analysis of extracellular vesicles isolated from porcine adipose tissue-derived mesenchymal stem/stromal cells. Sci. Rep..

[57] Galzie Z., Kinsella A.R., Smith J.A. (1997). Fibroblast growth factors and their receptors. Biochem. Cell Biol..

[58] Javerzat S., Auguste P., Bikfalvi A. (2002). The role of fibroblast growth factors in vascular development. Trends Mol. Med..

[59] Yun Y.-R., Won J.E., Jeon E., Lee S., Kang W., Jo H., Jang J.-H., Shin U.S., Kim H.-W. (2010). Fibroblast growth factors: Biology, function, and application for tissue regeneration. J. Tissue Eng..

[60] Bucan V., Vaslaitis D., Peck C.T., Strauss S., Vogt P.M., Radtke C. (2019). Effect of exosomes from rat adipose-derived mesenchymal stem cells on neurite outgrowth and sciatic nerve regeneration after crush injury. Mol. Neurobiol..

[61] Wang J., Liu S., Li J., Yi Z. (2019). The role of the fibroblast growth factor family in bone-related diseases. Chem. Biol. Drug Des..

[62] Ohbayashi N., Shibayama M., Kurotaki Y., Imanishi M., Fujimori T., Itoh N., Takada S. (2002). Fgf18 is required for normal cell proliferation and differentiation during osteogenesis and chondrogenesis. Genes Dev..

[63] Ford-Perriss M., Abud H., Murphy M. (2001). Fibroblast growth factors in the developing central nervous system. Clin. Exp. Pharmacol. Physiol..

[64] Murase S., Mosser E., Schuman E.M. (2002). Depolarization drives β-catenin into neuronal spines promoting changes in synaptic structure and function. Neuron.

[65] Strand N.S., Hoi K.K., Phan T.M., Ray C.A., Berndt J.D., Moon R.T. (2016). Wnt/β-catenin signaling promotes regeneration after adult zebrafish spinal cord injury. Biochem. Biophys. Res. Commun..

[66] Yin Z.-S., Zu B., Chang J., Zhang H. (2008). Repair effect of wnt3a protein on the contused adult rat spinal cord. Neurol. Res..

[67] Rodriguez J., Esteve P., Weinl C., Ruiz J.M., Fermin Y., Trousse F., Dwivedy A., Holt C., Bovolenta P. (2005). Sfrp1 regulates the growth of retinal ganglion cell axons through the fz2 receptor. Nat. Neurosci..

[68] Garcia A.L., Udeh A., Kalahasty K., Hackam A.S. (2018). A growing field: The regulation of axonal regeneration by wnt signaling. Neural Regen. Res..

[69] Park H., Poo M.M. (2013). Neurotrophin regulation of neural circuit development and function. Nat. Rev. Neurosci..

[70] Huang E.J., Reichardt L.F. (2001). Neurotrophins: Roles in neuronal development and function. Annu. Rev. Neurosci..

[71] Binder D.K., Scharfman H.E. (2004). Brain-derived neurotrophic factor. Growth Factors (ChurSwitz.).

[72] Zhao C., Deng W., Gage F.H. (2008). Mechanisms and functional implications of adult neurogenesis. Cell.

[73] Schäbitz W.-R., Schwab S., Spranger M., Hacke W. (1997). Intraventricular brain-derived neurotrophic factor reduces infarct size after focal cerebral ischemia in rats. J. Cereb. Blood Flow Metab..

[74] Oo T.F., Kholodilov N., Burke R.E. (2003). Regulation of natural cell death in dopaminergic neurons of the substantia nigra by striatal glial cell line-derived neurotrophic factor in vivo. J. Neurosci. Off. J. Soc. Neurosci..

[75] Zhang B., Wang M., Gong A., Zhang X., Wu X., Zhu Y., Shi H., Wu L., Zhu W., Qian H. (2015). Hucmsc-exosome mediated-wnt4 signaling is required for cutaneous wound healing. Stem Cells.

[76] Ahn S.Y., Park W.S., Kim Y.E., Sung D.K., Sung S.I., Ahn J.Y., Chang Y.S. (2018). Vascular endothelial growth factor mediates the therapeutic efficacy of mesenchymal stem cell-derived extracellular vesicles against neonatal hyperoxic lung injury. Exp. Mol. Med..

[77] Sun H., Hu S., Zhang Z., Lun J., Liao W., Zhang Z. (2019). Expression of exosomal micrornas during chondrogenic differentiation of human bone mesenchymal stem cells. J. Cell. Biochem..

[78] Wang X., Omar O., Vazirisani F., Thomsen P., Ekstrom K. (2018). Mesenchymal stem cell-derived exosomes have altered microrna profiles and induce osteogenic differentiation depending on the stage of differentiation. PLoS ONE.

